# 1358. A Real-World Study of the Burden of Illness and Treatment Patterns Among Patients with Uncomplicated Urogenital Gonorrhea in the United States

**DOI:** 10.1093/ofid/ofab466.1550

**Published:** 2021-12-04

**Authors:** Madison T Preib, Fanny S Mitrani-Gold, Ziyu Lan, Xiaoxi Sun, Ashish V Joshi

**Affiliations:** 1 STATinMED Research, Ann Arbor, MI, USA, Ann Arbor, Michigan; 2 GlaxoSmithKline plc, Collegeville, PA, USA, Chicago, Illinois

## Abstract

**Background:**

Gonorrhea (GC) is a major public health threat in the US. The Centers for Disease Control and Prevention (CDC) estimated direct healthcare costs of &271 million in 2018. CDC 2015 guidelines (applicable up to December 18, 2020) recommended cephalosporin plus azithromycin for GC. We used real-world data to assess patterns of inappropriate *or* suboptimal (IA/SO) or appropriate *and* optimal (AP&OP) antibiotic (AB) prescription (by CDC 2015 guidelines), and related healthcare costs, in US patients with uncomplicated urogenital GC (uUGG) diagnosed from July 1, 2013–June 30, 2018.

**Methods:**

A retrospective cohort study of IBM MarketScan data (commercial/Medicare claims) in patients ≥ 12 years old with uUGG. Eligible patients had an AB prescription ±5 days of uUGG diagnosis (index date) and continuous health-plan enrollment with ≥ 6 months’ baseline/≥ 12 months’ follow-up data. Patients with complicated urogenital GC were excluded. Patients were stratified by AB prescription (IA/SO or AP&OP; defined in **Table 1**) during the first uUGG episode (ie, within 30 days of index). Generalized linear models were used for multivariate analysis.

Table 1. Definitions of appropriateness of AB prescriptions

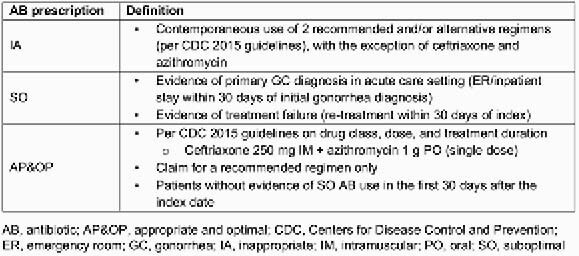

**Results:**

Of 2847 patients with uUGG (58.5% male), 77.1% had an IA/SO prescription (mostly due to IA AB class [~82.0%] and duration [24.0%]), while only 22.9% had an AP&OP prescription; uUGG episodes were more frequent with IA/SO (n=2386) than AP&OP (n=714) prescriptions during follow-up. Patients with IA/SO prescriptions had higher GC-related total adjusted costs per patient (PP) per index episode (&196) vs those with AP&OP prescriptions (&124, p < 0.0001; Figure). Patients with IA/SO prescriptions also had higher GCrelated total adjusted costs PP during follow-up (&220) vs those with AP&OP prescriptions (&148, p < 0.0001), mostly driven by higher outpatient ambulatory and emergency room (ER) adjusted costs with IA/SO (&148 and &71, respectively) vs AP&OP prescriptions (&129 and &12, respectively, p ≤ 0.0152; Figure). ER visits PP at index and during follow-up were higher with IA/SO vs AP&OP prescriptions (p < 0.0001; Table 2).

Figure. GC-related costs per patient with uUGG, stratified by appropriateness of AB prescription*

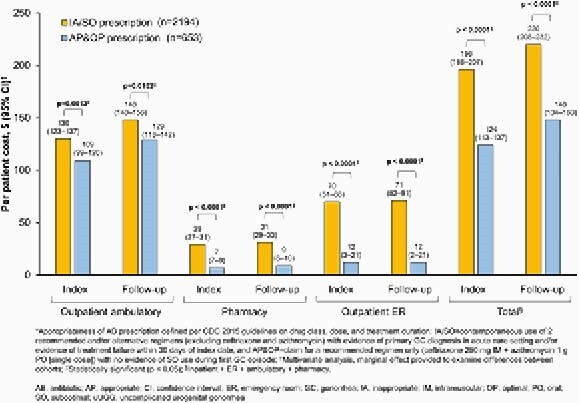

Table 2. GC-related HRU per patient with uUGG, stratified by AB prescription

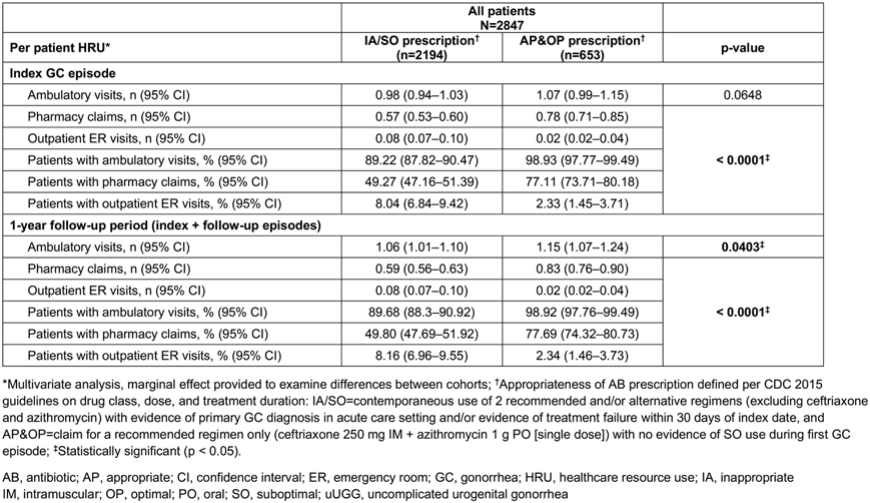

**Conclusion:**

Most patients with uUGG were not prescribed treatments in accordance with CDC 2015 guidelines. High IA/SO AB prescriptions and associated healthcare costs suggest an unmet need for improved prescribing practices for uUGG in the US.

**Disclosures:**

**Madison T. Preib, MPH**, **STATinMED Research** (Employee, Former employee of STATinMED Research, which received funding from GlaxoSmithKline plc. to conduct this study) **Fanny S. Mitrani-Gold, MPH**, **GlaxoSmithKline plc.** (Employee, Shareholder) **Ziyu Lan, MSc**, **STATinMED Research** (Employee, Employee of STATinMED Research, which received funding from GlaxoSmithKline plc. to conduct this study) **Xiaoxi Sun, MA**, **STATinMED Research** (Employee, Employee of STATinMED Research, which received funding from GlaxoSmithKline plc. to conduct this study) **Ashish V. Joshi, PhD**, **GlaxoSmithKline plc.** (Employee, Shareholder)

